# Using an Innovation Arena to compare wild-caught and laboratory Goffin’s cockatoos

**DOI:** 10.1038/s41598-020-65223-6

**Published:** 2020-05-26

**Authors:** Theresa Rössler, Berenika Mioduszewska, Mark O’Hara, Ludwig Huber, Dewi M. Prawiradilaga, Alice M. I. Auersperg

**Affiliations:** 1 Comparative Cognition, Messerli Research Institute, University of Veterinary Medicine Vienna, University of Vienna, Medical University of Vienna, Veterinaerplatz 1, 1210 Vienna, Austria; 2 Research Center for Biology, Indonesian Institute of Sciences, Jl. Raya Jakarta – Bogor, Km.46 Cibinong, 16911 Bogor, Indonesia; 3 Department of Cognitive Biology, University of Vienna, Althanstraße 14, 1090 Vienna, Austria; 4 Max Planck Institute for Ornithology, Eberhard-Gwinner-Straße, 82319 Seewiesen, Germany

**Keywords:** Behavioural ecology, Evolutionary ecology

## Abstract

The ability to innovate, i.e., to exhibit new or modified learned behaviours, can facilitate adaptation to environmental changes or exploiting novel resources. We hereby introduce a comparative approach for studying innovation rate, the ‘Innovation Arena’ (IA), featuring the simultaneous presentation of 20 interchangeable tasks, which subjects encounter repeatedly. The new design allows for the experimental study of innovation per time unit and for uncovering group-specific problem-solving abilities – an important feature for comparing animals with different predispositions and life histories. We applied the IA for the first time to investigate how long-term captivity affects innovative capacities in the Goffin’s cockatoo, an avian model species for animal innovation. We found that fewer temporarily-captive wild birds are inclined to consistently interact with the apparatus in comparison to laboratory-raised birds. However, those that are interested solve a similar number of tasks at a similar rate, indicating no difference in the cognitive ability to solve technical problems. Our findings thus provide a contrast to previous literature, which suggested enhanced cognitive abilities and technical problem-solving skills in long-term captive animals. We discuss the impact and discrepancy between motivation and cognitive ability on innovation rate. Our findings contribute to the debate on how captivity affects innovation in animals.

## Introduction

The ability to innovate is a significant aspect of a species’ adaptability to a changing environment. Studying innovative behaviour is thus key to understanding the evolution of cognitive flexibility in both, human and non-human animals^1,2^.

In general, animal innovation has been defined rather broadly as new or modified learned behaviour^3^, to discover and internalize new solutions to known or unknown problems^1^, or more specifically as ‘the discovery of a new behavioural interaction with the social or physical environment, tapping into an existing opportunity and/or creating a new opportunity’^4^. A single innovation does thus not unconditionally require complex cognition such as cause and effect understanding or planning. A novel problem can also be solved by exploration, which is dependent on an agent´s motoric repertoire, and basic cognitive processes such as attentional and/or inhibitory control, flexibility, memory and associative learning. Nevertheless, the rate at which birds and primates innovate correlates with residual brain size and multimodal integration of areas necessary for complex cognition^2,5–11^.

The innovative capacity of different groups is typically addressed by confronting subjects with a single or a small set of tasks and scoring their performances against one another (see review in^12^). Groups may differ on a species level or in other attributes, such as life history, age, sex or experience. Such approaches can be used to identify correlations of problem-solving performance with other measures, such as neophobia, exploration, or learning (e.g.,^13–15^). However, animals often have different approaches to specific problems depending on their morphological toolkit, their motivation and their exploration styles^16,17^. Different groups may outperform one another in different problems and it can be difficult to control for inherited predispositions or pre-experiences. Thus, individual problems may require different levels of innovative strength from different groups of animals (as discussed e.g., in^18,19^).

A way to counter these challenges is to present subjects with a set of several tasks^14,17,20–23^. Confronting animals with a series of consecutive problems targeting specific processes^24,25^ can be informative on differences in cognitive performance and may provide insight on their innovative capacity. However, it cannot be used to study how their innovation rate develops as innovation events (i.e., the emergence of innovations) over time cannot be measured in a controlled manner - an important step towards an understanding of how an animal actively adapts to environmental changes. Additionally, order effects of consecutive presentation of several tasks are difficult to control and their influence might vary between species.

A solution to this limitation is simultaneous presentation of task sets. An example of a task battery in which multiple problems are presented simultaneously rather than consecutively is the ‘Multi Access Box’ paradigm (MAB;^17^). It features four solutions to obtain a single reward in the centre of a cubic box. Subjects could use any solution from the start but after performing the same one repeatedly, it was blocked, forcing them to switch to another solution. This approach allowed for studying different innovative strategies (e.g. playful versus more target specific strategies such as ‘win-stay’) and task preferences due to ergonomical or motivational prerequisites in addition to ability^26^. Nevertheless, the MAB is also a limited tool for studying innovation rate itself: the single-reward-setup is restricted to one solution before the box requires rebaiting and therefore analysis of the time between single solutions is limited to a trial per trial basis.

Other studies used apparatuses with multiple access possibilities and multiple rewards to compare problem-solving^14,27,28^ and to allow the subjects to freely exhibit multiple innovative behaviours within one trial. The benefit for comparative studies of such an approach would be the possibility to measure the number of solutions found per time unit. However, this was not the focus of said studies as the subjects were allowed to interact with the apparatus only once which minimized the observed time frame to one trial. Furthermore, the set-up was also restricted to a maximum of four ergonomically distinct solutions or rewards. As acknowledged by the authors^27^, a broad comparative approach is most informative if the problems presented are greater in number and more diverse in their level of difficulty and can be applied to compare animals with different predispositions and morphological toolkits on a wider range. Some test groups might find the same amount of solutions to the tasks but in a longer period of time. Therefore, repeated exposure to an apparatus until no further solutions are found would allow to measure the emergent dynamics of innovation.

This paper has two main focuses: First to present a novel experimental design that allows us to study technical innovation rate comparatively between groups within and/or between different species. Secondly, we applied this approach to investigate whether long-term captivity affects the problem-solving abilities of Goffin’s cockatoos (*Cacatua goffiniana*; hereafter: ‘Goffins’), a model species for investigating physical cognition.

**Figure 1:**
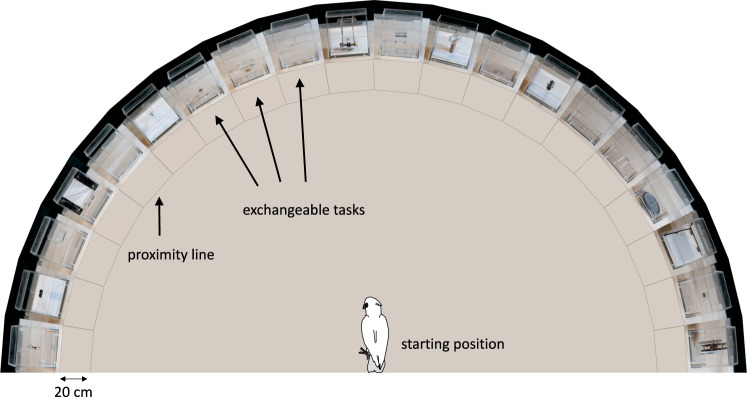
The Innovation Arena. Tasks arranged in a semi-circle; the positions of the 20 tasks were exchangeable. A proximity grid (20 cm in front of each box) is marked in dark brown. Dimensions not to scale.

**Figure 2:**
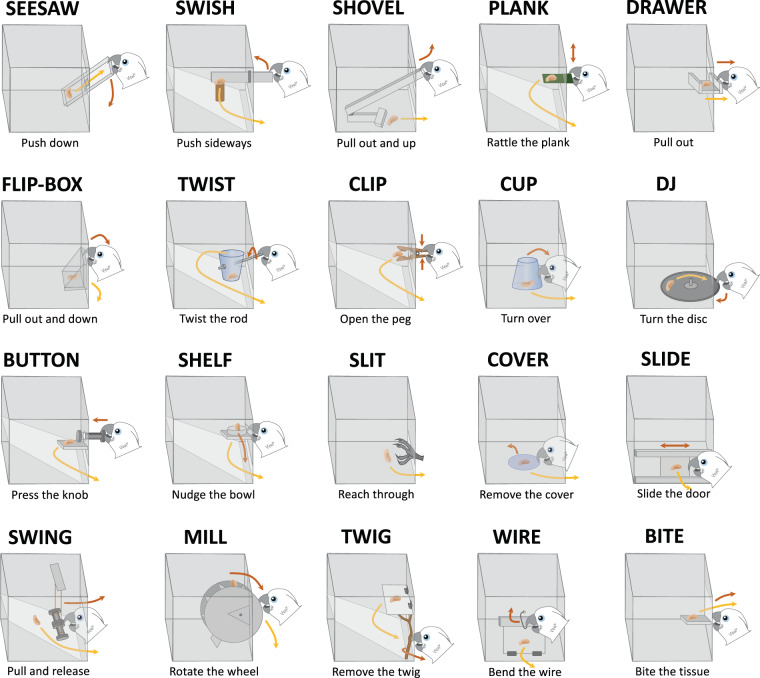
Tasks of the Innovation Arena with a corresponding description of the motoric action required for solving ( = reward; red arrows indicate directions of actions required to solve tasks; yellow arrows indicate reward trajectories). Tasks are arranged according to their mean difficulty (left to right, top to bottom).

We introduce an ‘Innovation Arena’ (from here forth, ‘IA’) as a new controlled approach for comparatively studying innovative behaviour and innovation rate. In the IA subjects were confronted with 20 different technical problems simultaneously, each of which was baited with a single preferred food reward (Fig. 1). This implementation of a broader spectrum of tasks creates more opportunities to elicit innovative behaviour and is more robust to the influence of single tasks. It further allows analyses of group comparisons in a more detailed manner and has the potential to uncover patterns, e.g. which kind of tasks are most difficult, or even not solvable, for a particular group. Through the simultaneous presentation, innovations can be freely exhibited by the subjects in their individually preferred order while allowing to measure the emergence of those innovations over time. The tasks were novel to all subjects, each requiring distinctive motoric actions with no more than one simultaneous contact point. To allow for future comparative studies including different, also distantly related, species, the tasks were carefully designed to be ergonomically solvable with beak, foot/claws or hands/fingers (Fig. 2). The tasks were arranged in a semi-circle and each subject received 20 min to retrieve as many rewards as possible. Before the next session of each individual, the IA was rebaited but the positions of the tasks were randomly interchanged. Each subject was tested repeatedly (once per test day) until it either: (a) did not solve an additional task in five consecutive sessions, or (b) did not solve any task in ten consecutive sessions. This approach allowed us to compare the number of solutions achieved as a function of time across sessions until no new innovations occurred as well as group-specific task difficulty levels.

We apply the IA for the first time to compare two groups of the same species but with different life histories and experiences, laboratory-raised and wild-caught. Some aspects of innovation, such as technical problem-solving (including tool use), seem to be enhanced in several species under long-term captive conditions, a phenomenon known as ‘captivity effect’ or ‘captivity bias’^29–35^. While short-term captivity may already boost performance through forced proximity or food deprivation^29,30^, long-term captivity has been proposed to directly impact cognitive abilities due to enculturation through the extensive exposure to artificial environments and objects as well as the proximity to and frequent interaction with humans (e.g.,^36,37^). The existing evidence concerning captivity bias on innovation is based largely on observational and often anecdotal data. There are relatively few attempts to directly compare problem-solving performance of wild and captive animals^27,28,31,32,37–39^ with only two studies on kea and one on hyenas targeting innovative problem-solving performance between wild and long-term captive animals. In both cases the wild subjects were free-ranging and tested in their natural habitat. While ultimately an important undertaking, such studies can be hard to interpret due to the lack of control over social and environmental distractors, as acknowledged by the respective authors^32^.

In this study, we investigate possible effects of long-term captivity on the innovative capacity in Goffin’s cockatoos. This parrot is an opportunist-generalist endemic to the Tanimbar Islands, a small archipelago in the Moluccas, Indonesia^40,41^. It has become an important avian model for investigating innovation and problem-solving (see review in^42^) as innovativeness is often linked to opportunism^5,15,43^ and avian technical problem-solving skills parallel higher primates (e.g.,^44,45^). Goffins can innovate sophisticated forms of tool use and manufacture^18,46,47^ despite not being dependent on tool-obtained resources in the wild^40,41^. However, all previous findings so far were exclusively based on a long-term laboratory population of Goffins which is problematic as innovativeness in captivity does not necessarily predict innovativeness in the wild^37^. Thus, we consider comparing innovative behaviour and innovation rate in laboratory-raised versus wild-caught Goffins an important next step and an ideal opportunity to apply the IA approach for the first time.

A captivity bias in problem-solving ability would imply an increased innovation rate in laboratory-raised subjects, particularly when considering human-made, artificial problems. Wild-caught birds should find fewer solutions, might show a preference for previously solved tasks across sessions, and have more problems in detecting novel affordances than laboratory birds.

## Results

### Qualitative analysis of apparatus-directed behaviour

Within both test groups (laboratory (‘Lab’) and wild-caught (‘Field’)) significant differences in motivation to interact with the apparatus were observed. The subjects were either very active and engaged with the apparatus readily and for an extended period of time or showed hardly any interest. If individuals did not touch any task within the first 3 min of a test session, their attention was guided towards the IA with food rewards. Individuals who required an implementation of this ‘motivational’ protocol at any point of this study (see Supplementary Methods for details) were classified as ‘unmotivated’, whereas individuals who did not require any motivational protocols were classified as ‘motivated’. The ratio of motivated to unmotivated subjects was higher in the Lab group (10 out of 11) than in the Field group (3 out of 8; Fisher´s exact test: *p* = 0.04).

### Quantitative analysis of apparatus-directed behaviour

A Bartlett’s test revealed significant correlations (𝜒^2^ = 1203.5, *df* = 15, *p* < 0.001) between the measured apparatus-directed behaviours. We therefore used a principal component analysis before inclusion to the model. It revealed two components which explained together 76.7% of the variance (Supplementary Table S2 shows PCA output). ‘Principal Component 1’ (PC1) entailed frequency of contact with tasks (baited and solved), duration of time in proximity and the number of tasks touched and explained 58.6% of the variance. ‘Principal Component 2’ (PC2) loaded negatively on number of tasks touched but not solved, positively on contact with already solved task and described 18.2% of the total variance. Post-hoc analysis revealed that the Lab group showed significantly higher levels of PC1 (*W* = 78, *p* < 0.001; see Supplementary Fig. S1) and PC2 (*W* = 77, *p* < 0.001) than the Field group.

All motivated individuals had mean levels above 0 for PC1 (range: 0.148–1.972), while values for unmotivated subjects ranged from −2.714 to −2.2, supporting our original classification (see Supplementary Table S1).

### Probability to solve

**Figure 3:**
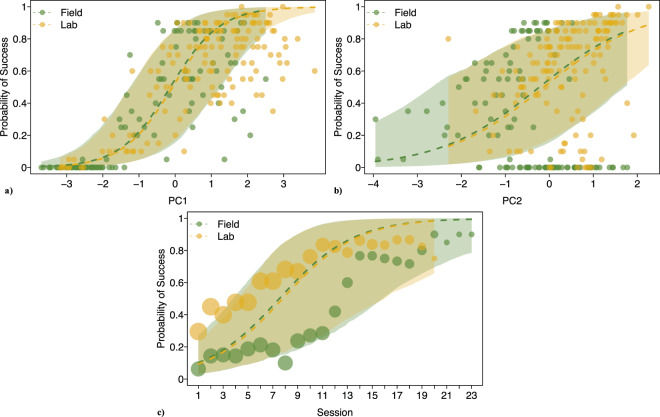
Influence of control predictors on probability to solve: (**a**) PC1, (**b**) PC2, (**c**) Session. Points show observed data, size of points indicates number of observations for each data point, dashed lines show fitted values of model and areas symbolize confidence intervals of model.

We used a Generalized Linear Mixed Model to investigate the probability to find solutions (see Methods for a detailed description). There was a significant combined impact of Group, PC1 and PC2 on the probability to solve (full-null model comparison: 𝜒^2^ = 29.64, *df* = 3, *p* < 0.001). We then dropped one term at a time from the model while controlling for others. This procedure reveals whether or not the dropped term (e.g., Group) has a significant influence on the probability to solve when all other measures are kept constant. We found no significant effect for Group (estimate = −0.089, *SE* ± 1.012, *z* = −0.09, *p* = 0.945). An increase in PC1 or PC2 significantly increased the probability to solve tasks (PC1: estimate = 2.713, *SE* ± 0.588, *z* = 4.61, *p* < 0.001; PC2: estimate = 0.906, *SE* ± 0.315, *z* = 2.87, *p* = 0.003). Additionally, we found a significant effect of Session: more tasks were solved in later than in earlier sessions (estimate = 1.719, *SE* ± 0.526, *z* = 3.27, *p* = 0.001) (see Fig. 3, Table 1 and Table 2 for model output).

**Table 1:** Fixed effects results of the model for probability to solve.

**term**	**estimate**	**SE**	**lower CI**	**upper CI**	**𝜒**^2^	**df**	**P**	**min**	**max**
(Intercept)^a^	0.196	1.209	−1.65	1.808				−1.064	1.358
Group.lab^a^	−0.089	1.012	−1.269	1.152	0.005	1	0.945	−0.869	1.485
PC1^b^	2.713	0.588	2.069	3.343	28.64	1	<0.001	2.256	3.249
PC2^b^	0.906	0.315	0.552	1.245	9.106	1	0.003	0.627	1.261
session^b^	1.719	0.526	0.819	2.546	6.303	1	0.001	1.434	1.982

^a^Dummy coded with group ‘Field’ being the reference category.

^b^z-transformed to a mean of 0 and a standard deviation of 1.

**Figure 4:**
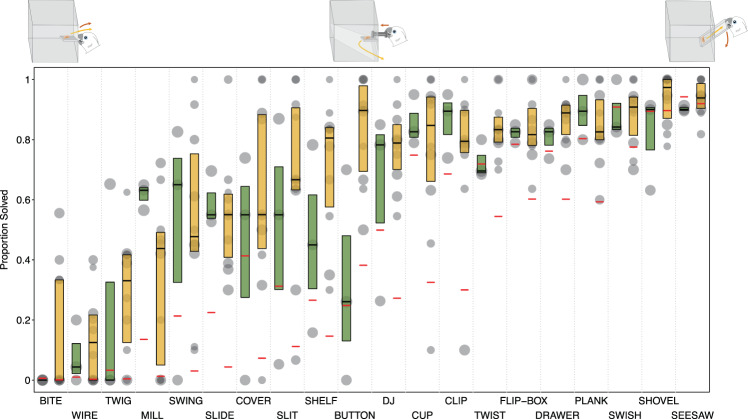
Observed data of motivated birds as well as fitted values of model per task and group: Boxplots show the proportion of successes per task for both groups (green = Field; orange = Lab). Bold horizontal lines indicate median values, boxes span from the first to third quartiles for motivated birds only (to improve visual clarity). Individual observations are depicted by points (larger points indicate more observations per data point). Red horizontal lines show fitted values. Included are illustrations of Bite, Button and Seesaw tasks (left to right).

Individual tasks seemed to be of varying difficulty (Fig. 4, Table 2) but overall, Lab and Field groups did not vary significantly in their success depending on the task (comparison of full model with reduced model lacking random slope of Group within Task: 𝜒^2^ = 7.589, *df* = 5, *p* = 0.18).

**Table 2:** Model estimates and rank (1–20) of task difficulty per group.

Task	Estimates		Rank	
	Field^a^	Lab^a^	Field	Lab
Bite	−5.19	−5.48	20	20
Button	0.16	2.12	**11**	**4**
Clip	0.82	0.37	*9*	*10*
Cover	−0.64	−1.63	14	14
Cup	1.00	0.32	**8**	**11**
DJ	0.39	0.59	*10*	*9*
Drawer	1.71	2.10	*6*	*5*
Flip-Box	1.75	1.98	*5*	*6*
Mill	−2.26	−3.49	17	17
Plank	1.84	1.94	**4**	**7**
Seesaw	3.58	4.29	1	1
Shelf	−0.49	0.00	12	12
Shovel	3.10	4.25	2	2
Slide	−1.29	−1.94	15	15
Slit	−0.56	−0.58	13	13
Swing	−1.56	−2.45	16	16
Swish	2.70	2.74	3	3
Twig	−3.59	−4.30	*18*	*19*
Twist	1.40	1.78	*7*	*8*
Wire	−4.43	−4.25	*19*	*18*

*Note*. Differences of more than 1 rank between groups are highlighted in bold; differences of 1 rank in italics.

^a^Dummy coded with Field group being the reference category.

Lower estimates indicate a lower probability to be solved.

Post-hoc analysis revealed a significant interaction of Group and Session (estimate = 2.924, *SE* ± 0.854, *z* = 3.423, *p* = 0.001; see Table 3 for an overview of all statistical tests and results).

**Table 3:** Summary of conducted statistical tests.

Test	Measure for	Result
Bartlett’s test	correlations of apparatus-directed behaviours	𝜒^2^ = 1203.5, *df* = 15, ***p*** < **0.001**
full vs. null model	combined influence of Group, PC1 and PC2	𝜒^2^ = 29.64, *df* = 3, ***p*** < **0.001**
***full model vs. reduced models***		
without fixed effect Group	influence of Group	estimate = −0.089, *z* = −0.09, *p* = 0.945
without fixed effect PC1	influence of PC1	estimate = 2.713, *z* = 4.61, ***p*** < **0.001**
without fixed effect PC2	influence of PC2	estimate = 0.906, *z* = 2.87, ***p*** = **0.003**
without fixed effect Session	influence of Session	estimate = 1.719, *z* = 3.27, ***p*** = **0.001**
without random slope of Group within Task	influence of Group in task difficulty	𝜒^2^ = 7.589, *df* = 5, *p* = 0.18
***post-hoc tests***		
full model + interaction term vs. without interaction term	influence of interaction Group * Session	estimate = 2.924, *z* = 3.423, ***p*** = **0.001**
Fisher’s exact test (group classification)	difference in ratio of motivated and unmotivated subjects per group	***p*** = **0.04**
Mann-Whitney *U*-test (group in PC1)	difference of PC1 between groups	*W* = 78, ***p*** < **0.001**
Mann-Whitney *U*-test (group in PC2)	difference of PC2 between groups	*W* = 77, ***p*** < **0.001**

*Note*. Significant *p*-values below the threshold of 0.05 are in boldface; All model comparisons are likelihood ratio tests.

## Discussion

Our study successfully introduced the IA as a new paradigm to compare innovative behaviour. It is, to our knowledge, the first study specifically targeting innovation rate per time unit in animals and the first systematically controlled direct comparison of problem-solving between captive-born and temporarily wild-caught animals. It yielded a number of interesting findings, with the most significant one being that long-term captivity does not seem to affect the Goffins’ overall *capacity* to innovate in the IA but rather their *motivation* to do so.

We measured apparatus-directed behaviours and used a PCA to extract principal components. PC1 was affected by task proximity and physical contact with the apparatus while PC2 loaded positively on contact with already solved tasks and negatively on the number of tasks touched but not solved. Such behaviours are commonly used as measures for motivation (reviewed in^12^). Post-hoc tests revealed that both components were considerably higher in laboratory-raised than in wild-caught subjects (Supplementary Fig. S1). Although a unified definition of motivation is mostly lacking, two types are commonly recognized (see^48,49^ for review). The first, extrinsic motivation, is driven largely by external influences, such as food rewards, while the other, intrinsic motivation, refers to motivation facilitated by gratification from the task itself or ‘interest’ (e.g.,^48,50^) which is largely driven by exploration and curiosity^51^. Note that a lack of interest does not mean that an animal also lacks the overall cognitive competency to find the appropriate solution. Nonetheless, intrinsic as well as extrinsic motivation is often one of several components underlying performance, both on a species^48,52,53^, as well as on an individual level^13,14,54–59^.

The difference in subjects’ motivation between the two groups was clearly expressed in ratio rather than in degree: all six unmotivated birds (5 wild-caught, 1 laboratory) rarely approached the setup or interacted with the tasks (see Supplementary Table S1). In contrast, the remaining birds (3 wild-caught and 10 laboratory) consistently maintained their interest in the setup (Supplementary Fig. S2) and discovered a similar number of solutions at the same rate (Supplementary Fig. S3) despite being presented with a highly complex, artificial and novel setup. If any cognitive components, aside from motivation, required to find solutions in the IA were fundamentally different in wild-caught versus laboratory-raised birds, we would have expected the motivated wild-caught birds to perform at a different rate than the laboratory-raised birds.

In fact, when motivation was controlled for in our model, group identity, i.e., being either laboratory-raised or wild-caught, did not predict the probability of finding solutions to the technical problem-solving tasks in the IA (Fig. 3, Table 1 for estimates). This suggests that the cognitive capacity required for technical problem-solving in this species does not seem to be solely an artefact of the captive lifestyle or experimental history. Note, however, the observed interaction of group and session: Group identity seemed to have more impact in earlier sessions (Fig. 3c), which might be largely due to unmotivated birds.

Our results thus suggest that motivation is the main, if not the sole, cause for the differences observed. In other words, it is plausible that both wild-caught and laboratory-raised birds *can* (have the general capacity) perform at a similar level within the IA if they *want to* (are motivated to interact with the tasks). A captive life history does not seem to influence any other aspects of the overall cognitive capacity required to innovate in the IA.

Our findings contrast with previous literature, which suggested enhanced cognitive abilities and technical problem-solving skills in captive animals as a consequence of increased free time and energy as well as exposure to human environments and contact (e.g.,^1,29,32,36^). Whereas this may indeed be true for some species, particularly those closely related to humans, it may not apply to all. In fact, we believe it is possible that a long-term laboratory environment, even one featuring daily enrichment protocols, may fail to offer a greater cognitive challenge for an island dwelling parrot, such as the Goffin, than their natural habitat. The Tanimbar Islands provide a variety of feeding sources, including hard shelled, underground, patchy and/or seasonal items that are opportunistically exploited by the birds^40,41^. The arguably less predictable environment of wild Goffins coupled with their opportunity to encounter a greater variety of different problems and situations might have facilitated their flexibility^60^. Such opportunism is likely to allow the motivated wild-caught birds to swiftly adjust their problem-solving abilities to novel and artificial extractive foraging tasks. However, further studies are required as until now only a handful of studies have directly compared wild and captive-born groups of the same species in problem-solving performance under the same controlled conditions^32,54^.

Concerning the difference in motivation, there are several theoretical explanations for the observed greater proportion of motivated laboratory-raised versus wild-caught Goffins, some of which are less likely than others. The wild-caught birds did not seem to have problems seeing the reward through the acrylic glass because the vast majority of unmotivated birds solved at least one task during the course of this study. Furthermore, it is unlikely that different levels of neophobia substantially influenced subjects’ motivation. Although we cannot fully exclude residual neophobic reactions, we followed a detailed habituation protocol (see Supplementary Methods) to minimize its effect. Before test sessions started, all birds consumed a food reward from the top of each IA task while being individually separated.

It is also unlikely that the discrepancy in motivation was caused to a large degree by different levels of extrinsic motivation resulting from different types of food rewards: We used cashews for laboratory-raised and dry corn for wild-caught birds. While a qualitative difference of cashew over corn could in some cases impact motivation, here, the food rewards were carefully chosen according to the animals’ top foraging preferences. From all food items offered from within their known feeding repertoire, laboratory-born birds showed the highest preference for cashew nuts and wild-caught birds for corn (see Supplementary Methods for details). All wild-caught subjects immediately started consuming their rewards throughout habituation and also consumed the corn placed at the start position at the beginning of each test session.

In contrast, the observed differences in ratio of motivated birds might be explained by different outcome expectancies. Laboratory-raised birds that participated in many problem-solving tasks^18,44,46,61^ may have developed an expectancy to gain food through apparatus interaction, while the wild-caught birds were naïve to such tasks prior to the start of testing. Various expectancy theories on motivation (e.g.,^62–66^) predict that the expectation of an individual, i.e., the perceived likelihood of an occurrence, is influenced by past feedback and affects persistence during task acquisition. Thus, the unmotivated wild-caught birds may not have been as routinely interested in the test setup as a typical laboratory-raised bird would have been. Additionally, many captive-held animals, including a variety of parrot species, show the phenomenon of contra-freeloading, i.e., they willingly put effort into obtaining food (e.g., from an apparatus) although freely available food is simultaneously accessible^67,68^. The laboratory subjects are usually very eager to participate in experiments which suggests that tests might be considered as foraging enrichment substituting natural challenges. Nevertheless, one subject from the laboratory group showed similar low motivation to interact with the IA as the five wild-caught birds, suggesting that expectancy based on experience and contra-freeloading cannot fully explain observed difference in interest even though it might influence the proportion of motivated subjects.

Despite the extensive problem-solving pre-experience in laboratory-raised birds, both wild-caught and laboratory birds seemed to encounter similar overall difficulties with similar task types (see Fig. 4 and Table 2 for estimates and ranks of task complexity). Subjects of both groups performed better at tasks requiring a non-repetitive movement which was independent of the reward release mechanism (lateral slash, pulling, pushing, shoving, e.g., ‘Seesaw’, ‘Shovel’, ‘Swish’ or ‘Drawer’; see Fig. 4 and Table 2). Tasks that included repetitive actions, such as biting through toilet paper or turning a mill, seemed to cause more difficulties, however turning a disk in the ‘DJ’ task did not. In contrast to the DJ, in both the ‘Mill’ and the ‘Bite’ task, the food was possibly out of the subject’s sight during the manipulation itself, suggesting that confounded visibility of the reward during the manipulation may have also affected performance^69^. Less clearly structured tasks, in which the functional mechanism was not positioned directly at the reward but was slightly displaced (e.g. ‘Wire’ and ‘Twig’), also proved difficult for the subjects. Although we did not find a significant difference in group on overall task difficulty, laboratory birds tended to perform better in the ‘Button’ task than wild-caught birds (Fig. 4, Table 2). This task required the subjects to press a bolt to release the reward. In this singular case, experimental experience seemed to be of advantage. All laboratory-raised birds had previously participated in experiments involving the use of stick tools to push a reward^46,47,61,70,71^ whereas in their natural habitat wild Goffins are unlikely to require such motor patterns.

Qualitatively, the birds used different techniques to solve tasks. One example is the Bite task where toilet paper was attached with big clips on both sides and held in place by smaller clips at the bottom. Subjects discovered three different non-exclusive solutions to solve this task: They shredded the material with repetitive biting actions, pulled the paper laterally out of the big clips, or removed smaller clips which subsequently allowed them to push the paper inside and access the reward. Individuals used multiple techniques – often in a combined manner – signifying that Goffins do not seem to persistently remain with previously learned motor routines for the same task^18^. The ‘Clip’ task was mostly solved by applying pressure simultaneously to both sides of the clips but some subjects would position their beak at the side of the aluminium coil distal to the reward and pull or push the sides in opposing vertical directions. The laboratory birds also opened the Wire task in several instances by removing the window hinges (which were closer to the reward) instead of unbending the wire, suggesting a proximity-based innovative strategy possibly due to a conflict in subjects’ attentional focus^72^. Both wild-caught and laboratory-raised birds utilized both beaks and feet, particularly in tasks that required insertions. Parrots have highly sensitive soles containing Herbst corpuscles^73^ that can be used for haptic exploration and problem-solving (e.g.,^69^).

In summary, our study underlines both the feasibility as well as the informative value of an IA approach for direct comparisons of innovation rate. We were able to compare the identity and number of tasks solved across sessions by wild-caught and laboratory-raised groups as well as the difficulties encountered with specific task features. Notably, a laboratory-raised life history did not seem to affect the cognitive processes required for high rate innovation in the IA aside from motivation which suggests the lack of an overall cognitive shift towards enhanced problem-solving abilities in laboratory-raised versus wild-caught Goffins. However, more laboratory-raised birds were interested in the problem-solving setup than wild-caught birds. Highly controlled comparisons with identical tasks on wild and laboratory-raised populations of the same species are crucial for and enriching to the interpretation of experiments conducted under long-term laboratory conditions^54^.

At this stage, we can only speculate whether the observed difference in motivation is due to life in long-term captivity or due to experimental history. A fruitful avenue for future research might therefore be to test experimentally naïve but laboratory-raised birds using the IA. In order to study the role of the Goffins’ natural habitat, a next step may include comparisons of innovation rate between the Goffins and closely related non-island Corella species. Another important future direction are interspecific comparisons that additionally include more distantly related species, such as primates, using the IA. Ultimately, comparative investigations of animal innovation enhance our understanding of the evolution of problem-solving in various taxa.

## Methods

### Subjects

We tested 11 adult laboratory (four females; seven males; 6–10 years of age) and eight wild-caught Goffins (six females; two males; age unknown) between March and December 2017. Laboratory-raised birds were purchased as juveniles from certified European breeders and housed at Goffin Lab Goldegg, Austria. Wild birds were caught on the Yamdena island in the Tanimbar archipelago, Indonesia, and kept temporarily at Goffin Lab Tanimbar field station (see Supplementary Methods and Table S1 for a detailed description of subjects, housing, experimental history, and the capture-release procedure).

### Ethics

The study on the laboratory subjects was approved by the Ethics and Animal Welfare Committee of the University of Veterinary Medicine Vienna in accordance with good scientific practice guidelines and national legislation. As our experiments were purely appetitive and strictly non-invasive, they were classified as non-animal experiments in accordance with the Austrian Animal Experiments Act (TVG 2012). The field research in Indonesia was approved by the Ministry of Research, Technology and Higher Education (RISTEK) based on a meeting by the Foreign Researcher Permit Coordinating Team (10/TKPIPA/E5/Dit.KI/X/2016) who granted the permits to conduct this research to M.O. (410/SIP/FRP/E5/Dit.KI/XII/2016) and B.M. (411/SIP/FRP/E5/Dit.KI/XII/2016).

### Apparatus

The Innovation Arena (see Fig. 1) consisted of 20 acrylic glass boxes (base: 15 cm ×16 cm ×17.51 cm; height: 16 cm) which could be inter-changeably arranged in a semicircle (distance from central point: approx. 1 m) on a platform. The top surfaces of each box served as lids which could be opened for baiting and were secured by long bars placed across multiple boxes during testing. The bases were screwed directly on the wooden platforms to keep each box in place, while the rest of the cuboid could be placed on any of the 20 positions. Each box constituted a different technical problem task requiring various motoric actions to solve (see Fig. 2). All tasks were novel to all subjects. The positions of tasks were randomly re-arranged for each session (for the Lab group randomization was restricted to no task being at the same position twice per subject).

### Procedure

To control for different levels of neophobia subjects were first habituated to the apparatus (see Supplementary Methods and Table S1 for details). In test sessions each task was baited before individuals entered the test compartment voluntarily and were allowed 20 min to solve as many tasks as possible (rationale: 1 min per task). If a subject showed behavioural signs of agitation, the session was aborted. We applied a motivational protocol (see Supplementary Methods) if a subject did not touch any tasks within 3 min to enhance motivation. For each session task positions were reassigned and all tasks were rebaited. Testing was repeated until a subject either: (a) did not find solutions to additional tasks in 5 consecutive sessions, or (b) did not solve any task in 10 consecutive sessions. We used small pieces of cashew nuts as rewards for laboratory birds and dried corn for wild-caught subjects. Both rewards were identified as the food with highest preference value from a variety of available options (see Supplementary Methods) and were only provided for testing purposes. Due to the fact that wild Goffins fed substantially longer on dry corn kernels than laboratory birds fed on small cashew pieces, the timer during test sessions in the Field group was paused if feeding on a corn kernel has exceeded 3 sec. Timing was resumed once the feeding has stopped. Laboratory birds never fed longer than 3 sec on one reward. All sessions were video-recorded by a wide-angle camera (Goffin Lab Goldegg: Dahua DH-SD22204T-GN, Goffin Lab Tanimbar: GoPro Hero 3 White) mounted at the ceiling above the IA.

### Behavioural coding

Videos were analysed using Behavioral Observation Research Interactive Software (BORIS; version 6.0.5)^74^. We coded which tasks were touched, which tasks were solved, and apparatus-directed behaviours (see Supplementary Table S4 for a detailed coding protocol).

### Statistical analysis

#### Principal component analysis

We used multiple variables to measure apparatus-directed behaviours: the number of contacts with baited (‘BaitedContact’) and solved tasks (‘SolvedContact’), time spent within the 20 cm proximity grid of the tasks (‘GridTime’), latency to approach within 20 cm (‘LatencyGrid’), the number of tasks touched (‘TasksTouched’) and the number of tasks touched but not solved (‘TouchedNSolved’). A Bartlett’s test revealed significant correlations (𝜒^2^ = 1203.5, df = 15, p < 0.001). We therefore used a Principal Component Analysis with orthogonal rotation to avoid issues of collinearity in the model fitted later. We inspected their distribution and log transformed ‘LatencyGrid’ to avoid influential cases beforehand. It resulted in two components (PC1 and PC2) being above Kaiser´s criterion^75^ of Eigenvalues above 1 which were thus included in the model as covariates.

#### Probability to solve

We used a Generalized Linear Mixed Model with binomial error structure and logit link function^76^ to analyse the effect of ‘Group’ (Field vs. Lab) on the probability to solve a task and included the number of session (‘Session’) and the two components resulting from the PCA as control predictors. Prior to fitting the model, we *z*-transformed all covariates to a mean of zero and a standard deviation of one to achieve easier interpretable estimates^77^. Group was manually dummy coded and centered prior to inclusion. We included random intercepts for Subject and Task in addition to factors combining Subject and Task (‘Subj.Task’) as well as Subject and Session (‘SessionID’) in the model. Due to the low number of males in the Field group we did not include sex as a factor in the model. However, for interested readers a graph illustrating the collected data can be found in Supplementary Fig. S4. The model entailed random slopes within Task (PC1, PC2, Session, and Group), Subject (PC1, PC2, Session) and the combined factor of Subject and Task (PC1, PC2, Session). After fitting the model, we confirmed that none of the model assumptions were violated (see Supplementary Statistical Analysis for details). To avoid ‘cryptic multiple testing’^78^ we first compared the full model with a null model, which comprised the same random effects structure but was lacking the fixed effects group, PC1 and PC2. Only then individual predictors were tested. To assess whether there was a difference in task difficulty between groups we compared our full model with one lacking the random slope of Group within Task. For all comparisons we used likelihood ratio tests. Task difficulty was assessed by the model estimates for each task. The lower the estimate of each task, the less likely it was to be solved. Our sample encompassed 580 observations per estimated effect (5 fixed effects, 4 random effects) from 19 individuals (8–23 sessions each). The total number of successes amounted to 2,509.

#### Post-hoc tests

The results of the control predictor session (Fig. 3, Table 1) suggested a possible interaction of Group and Session. For this reason, we added the interaction term to the model and tested significance using a likelihood ratio test, post-hoc. We further inspected the difference between groups for PC1 and PC2 using Mann-Whitney *U*-tests and compared ratios of ‘motivated’ and ‘unmotivated’ birds per group with Fisher´s exact test, post-hoc.

#### Implementation

All statistical analyses were performed in RStudio^79^ (version 1.1.453) using the software R^80^ (version 3.5.1). In addition to the base R, we used the following packages: ‘rela’^81^ (version 4.1), ‘lme4’^82^ (version 1.1–21),‘car’^83^ (version 3.0–3), ‘ggplot2’^84^ (version 3.2.1.) and ‘cowplot’^85^ (version 1.0.0; see Supplementary Statistical Analysis for more details).

## Supplementary information

Supplementary Information.

Supplementary Movie S1.

Supplementary Movie S2.

Supplementary Tables.
