# Supplementary material for: Using an Innovation Arena to compare wild-caught and laboratory Goffin’s cockatoos
Source: Sci Rep. 2020 May 26;10:8681. doi: 10.1038/s41598-020-65223-6 (PMC7250841; doi:10.1038/s41598-020-65223-6)
Supplement: Supplementary file 1 — Supplementary Information. [file 41598_2020_65223_MOESM1_ESM.docx]

Using an Innovation Arena to compare wild-caught and laboratory Goffin´s cockatoos

Theresa Rössler^1,3,5^, Berenika Mioduszewska^1,4,5^, Mark O’Hara^1,2^, Ludwig Huber^1^, Dewi M. Prawiradilaga^2^, Alice M.I. Auersperg^1^

1Comparative Cognition, Messerli Research Institute, University of Veterinary Medicine Vienna, University of Vienna, Medical University of Vienna, Veterinaerplatz 1, 1210, Vienna, Austria. 2Research Center for Biology, Indonesian Institute of Sciences, Jl. Raya Jakarta – Bogor, Km.46 Cibinong, 16911, Bogor, Indonesia. 3Department of Cognitive Biology, University of Vienna, Althanstraße 14, 1090, Vienna, Austria. 4Max Planck Institute for Ornithology, Eberhard-Gwinner-Straße, 82319, Seewiesen, Germany. 5These authors contributed equally: Theresa Rössler and Berenika Mioduszewska. ✉e-mail: theresa@roessler.in; berenika.mioduszewska@vetmeduni.ac.at

Supplementary Materials

(1) Methods

(2) Statistical Analysis

(3) Results

(4) Figures

Fig. S1. Difference in the principal components between groups

Fig. S2. Values for PC1 per session for each individual.

Fig. S3. Overview of number of tasks solved per session for each individual.

Fig. S4. Observed proportion solved per group and sex.

(5) References for Supplementary Materials

(6) Further Electronic Supplementary Materials

Movie S1. Innovation Arena. Goffin Lab Tanimbar

Movie S2. Innovation Arena. Goffin Lab Goldegg

Supplementary Tables.xlxs

Dataset S1. InnovationArena

SUPPLEMENTARY MATERIALS

**(1) Methods**

**Housing of Subjects**

Both study groups (Field and Lab) were kept in social groups in enriched aviaries (Goffin Lab Goldegg: group size: 13–16; aviary indoors: 45 m^2^ ground area, 3–6 m high; aviary outdoors: 150 m^2^ ground area, 3–5 m high; Goffin Lab Tanimbar: group size: 20; aviary and test room: 50 m^2^ ground area, 2–3 m high). Enrichment for the wild-caught population consisted of natural vegetation of shrubs, trees, lianas, dry tree trunks, perches as well as daily fresh jungle foliage. Laboratory birds were offered a variety of perches, trees, parrot toys and cardboard. Both groups were provided with bathing opportunities, water and food (fresh and dried fruit, vegetables, a variety of seeds and minerals) ad libitum. The highest preferred food item (see below) was only used during experiments. Birds were individually marked with coloured foot rings and could be visually isolated from the group during testing in order to avoid social learning.

The animal keeping conditions for the Goldegg laboratory complied with the species-specific guidelines for parrots provided by the Austrian Animal Protection act. Furthermore, the keeping facility and the animals were registered at the local Animal Welfare Bureau (Bezirkshauptmanschaft St Pölten, NÖ). The housing conditions in the field complied with both Austrian Animal Protection act, husbandry guidelines for Cacatua spp. ^1^, as well as guidelines and ethical considerations for housing and management of Psittacine birds used in research ^2^.

**Food Preference Test**

In an earlier study we identified cashew nuts as the most desired food item for laboratory birds out of 20 different food types ^3^, which we therefore used in the present study as test rewards. In the field the availability of potential reward food was limited to dry corn and fresh peanuts. All subjects had a strong and stable preference for corn which also constitutes part of their natural diet ^4,5^.

**Experimental History**

All laboratory birds had previously participated in a number of experiments, mostly on tool use ^3,6–12^ and other technical problem-solving tasks involving the use of acrylic glass apparatuses ^13^, however not in the context presented in this study. For the field subjects this was their first and only experimental experience. All tasks included in the IA were novel to all individuals of both groups.

**Capture-release Procedure**

The Goffin Lab Tanimbar research field station in Indonesia featured an aviary which allowed controlled capture-release experiments. All necessary permits were obtained prior to capturing subjects in the field which included Letter of Research Permit (number: 6D/TKPIPA/FRP/SM/VI/2015) from the Indonesian Ministry of Research, Technology and Higher Education as well as Maluku Forestry Office (BKSDA Maluku) permit (number: S.563/IV-K.30/Tek/2015). Wild Goffins were captured using a nylon leg noose mounted on crops in close vicinity to the capture-release aviary. Active traps were monitored from a nearby hidden location and trapped birds were immediately collected and processed at the aviary: body measurements were taken, health status was checked, and a permanent LIPI ID numbered ring and a temporary colour ring for individual identification in the aviary were attached to legs. Once testing was concluded, all birds were subjected to a pre-release health check and their colored rings were removed. The release was performed according to a ‘soft release’ method ^14^, without any handling during the release itself in order to minimize stress (as directed by the International Union for Conservation of Nature guidelines; IUCN/SSC ^15^): the wall of the test room was opened and birds were free to leave at their own volition. Post-release support included food provided on top of the aviary for a week, however no Goffins returned to feed. Post-release, the birds were monitored for a week during which time they were observed to forage on wild fruit and to disperse from the general vicinity of the aviary.

**Habituation**

**Phase I (IA without tasks).** We habituated the subjects to the apparatus without the front parts of each box (no functional parts of the tasks). This was conducted in the following order: group sessions inside the aviaries, group sessions inside the testing compartments, small groups in the testing compartment, and finally individually in the test compartments. Before the subjects entered the testing area, each box was baited with a reward in the centre. This phase lasted until each individual consumed all 20 rewards in 3 consecutive sessions lasting 10 min for the Lab group and 20 min for the Field group. Wild birds fed substantially longer on dry corn kernels than laboratory birds fed on small cashew pieces (if a bird was feeding for longer than 3 sec in a test session following the habituation, the timer was paused for the duration of feeding).

**Phase II (task elements).** To reduce neophobic reactions to the task elements we placed rewards on opaque elements which were part of the functional mechanisms of the tasks and offered them to the subjects to feed from. Habituation criterion required all subjects to be comfortably feeding on rewards spread on top of the detached IA task elements.

**Phase III (IA with tasks attached).** Subjects were confronted with the IA with all parts of the task mechanisms attached and functional. To control for neophobia towards fully assembled tasks, rewards were placed in full view of the subjects on top of each box on the edge closest to the bird. Subjects were only allowed to feed on the provided rewards, not to manipulate the tasks. Habituation was completed once each bird consumed all 20 rewards within one session (10 min for Lab group, 20 min for Field group).

**Motivational Protocol**

If a subject did not touch any task within 3 min *and* was not agitated, a motivational protocol was included within the same session. If at any point the subject started to be agitated, the session was immediately terminated and the subject was released. Motivational protocols were necessary to implement for a total of 6 birds (5 wild-caught, 1 lab) and consist of 4 steps:

**Step I** Rewards were placed on top of the task boxes (a total of 3 rewards: 1 in the middle and 2 on the side boxes). If the subject started touching tasks, a test session was resumed (20 min session duration from that point).

**Step II** If the bird did not touch any task within 3 min, all rewards placed on the boxes were moved on the approach grid line with 2 more rewards added (a total of 5 rewards on the line). If the subject started touching tasks, a test session was resumed (20 min session duration from that point).

**Step III** If the bird did not touch any task, all 5 rewards were placed in a small pile at the start position. If the subject started touching tasks, a test session was resumed (20 min session duration from that point).

**Step IV** If the bird did not touch any task, a small handful of rewards was added at the start position, the test session was terminated and a 10 min habituation session was commenced. After the 10 min have passed, the subject was released.

**(2) Statistical Analysis**

**Statistical Analysis**

**Model assumptions.** After fitting the model, we confirmed that none of the model assumptions were violated: we found that it was not overdispersed (dispersion parameter = 0.31) and normal distribution of ‘Best Linear Unbiased Predictors’ (BLUPs) was assessed visually (see ^16,17^). We further did not find any reason for concern with regard to collinearity of predictors when calculating ‘Variance Inflation Factor’ (VIF) ^18^ for a standard linear model excluding the random effects (VIF of 1.999 for Group, 1.583 for PC1, 1.48 for PC2, 1.159 for Session). Model stability was assessed by comparing estimates obtained from the model based on all data with estimates obtained from models in which the levels of random effects were excluded one at a time ^19^. All estimates proved to be fairly stable (see main article Table 1).

**Implementation.** The R software package ‘rela’ ^20^ (version 4.1) was used to identify correlations among predictors and ‘prcomp’ function for the PCA. The generalized mixed model was fitted by the function ‘glmer’ ^21^ (package ‘lme4’, version 1.1–21) using the optimizer ‘bobyqa’. The linear mixed models were fitted with the function ‘lmer’ from the same package. To assess ‘Variance Inflation Factors’ the function ‘vif’ of the package ‘car’ ^22^ (version 3.0–3) was used. Model stability was assessed using a function provided by Roger Mundry. Confidence intervals were derived applying the function ‘bootMer’ of the package ‘lme4’, using 1,000 parametric bootstraps for both fixed effects and random effects. Functions ‘anova’ and ‘drop1’ (argument ‘test’ set to ‘Chisq’) were used for likelihood ratio tests. Plotting the results was conducted with the package ‘ggplot2’ ^23^ (version 3.2.1) and ‘cowplot’ ^24^ (version 1.0.0) besides base R software packages.

**(3) Results**

**Repositioning on Principal Components.** In the Lab group, if a subject left the IA and became preoccupied with something else in the test compartment (e.g., flying to the ground) the experimenter repositioned the bird by hand at the start position on the test table (see Supplementary Table 3 for the coding protocol). To investigate whether this procedure (‘Repositioning’) influenced the difference in PCs between Lab and Field groups, we fitted a linear mixed model with PC1 as response variable and number of repositions as predictor. Additionally, we included PC2 and Session as control predictors. To avoid pseudoreplication we included subjects as random intercept with random slopes of PC2, Session and Repositioning. Prior to fitting the model, we square-root transformed PC1 and Repositioning. We used *z*-transformations for all control predictors and random effects. After fitting the model, we inspected whether any assumptions for linear models were violated. Visual inspection revealed normally distributed and homogeneous residuals (QQ-plot of residuals ^18^ and residuals plotted against fitted values ^25^). Collinearity, determined for a standard linear model lacking the random effects, appeared to be no issue (maximum Variance Inflation Factor: 2.243 for Session). Model stability was assessed as above and revealed fairly stables results (Supplementary Table 4). The same procedure was applied to PC2, the only difference being that PC2 and Repositioning did not need to be transformed to fit the model. Our sample comprised 143 occurrences of repositioning obtained from 11 subjects of the Lab group. We did not find any effect of Repositioning on PC1 (full-null model comparison: 𝜒^2^ = 0.782, *df* = 1, *p* = 0.377) nor on PC2 (full-null model comparison: 𝜒^2^ = 1.207, *df* = 1, *p* = 0.272). See Supplementary Table 5 and Table 6 for model output.

**(4) Figures**

**Fig. S1: Difference in the principal components between groups**. Bold horizontal lines indicate median values, boxes span the first to third quartiles.

**b)**

**a)**

**Fig. S2: Values for PC1 per session for each individual.** a) Field group, b) Lab group. Red lines = female; blue lines = male. Unmotivated birds are marked with grey background.

**Fig. S3: Overview of number of tasks solved per session for each individual.** a) Field group, b) Lab group. Red lines = female; blue lines = male. Unmotivated subjects are marked with grey background.

**Fig. S4: Observed proportion solved per group and sex.** a) All subjects, b) Motivated birds only. Bold horizontal lines indicate median values, boxes span from the first to third quartiles. Individual observations are depicted by points (larger points indicate more observations per data point).

**(5) References for Supplementary Materials**

1. O’Brien, J. *EEP Husbandry Guidelines for Cacatua spp. (2nd Edition)*. (2007).

2. Kalmar, I. D., Janssens, G. P. J. & Moons, C. P. H. Guidelines and Ethical Considerations for Housing and Management of Psittacine Birds Used in Research. *ILAR Journal* **51**, 409–423 (2010).

3. Laumer, I. B., Bugnyar, T. & Auersperg, A. M. I. Flexible decision-making relative to reward quality and tool functionality in Goffin cockatoos *(Cacatua goffiniana)*. *Scientific Reports* **6**, (2016).

4. Mioduszewska, B. *et al.* Notes on ecology of wild Goffin´s cockatoo in the late dry season with emphasis on feeding ecology. *Treubia* **14**, 85–102 (2018).

5. O’Hara, M. *et al.* Extraction without tooling around — The first comprehensive description of the foraging- and socio-ecology of wild Goffin’s cockatoos *(Cacatua goffiniana)*. *Behaviour* **1**, 1–30 (2018).

6. Auersperg, A. M. I., Borasinski, S., Laumer, I. B. & Kacelnik, A. Goffin’s cockatoos make the same tool type from different materials. *Biology Letters* **12**, 20160689 (2016).

7. Auersperg, A. M. I., Köck, C., O’Hara, M. & Huber, L. Tool making cockatoos adjust the lengths but not the widths of their tools to function. *PloS one* **13**, e0205429 (2018).

8. Auersperg, A. M. I., Köck, C., Pledermann, A., O’Hara, M. & Huber, L. Safekeeping of tools in Goffin’s cockatoos, *Cacatua goffiniana*. *Animal behaviour* **128**, 125–133 (2017).

9. Auersperg, A. M. I. *et al.* Social transmission of tool use and tool manufacture in Goffin cockatoos (Cacatua goffini). *Proceedings of the Royal Society B: Biological Sciences* **281**, 20140972–20140972 (2014).

10. Auersperg, A. M. I., Szabo, B., von Bayern, A. M. P. & Kacelnik, A. Spontaneous innovation in tool manufacture and use in a Goffin’s cockatoo. *Current Biology* **22**, R903–R904 (2012).

11. Beinhauer, I., Bugnyar, T. & Auersperg, A. M. I. Prospective but not retrospective tool selection in the Goffin’s cockatoo *(Cacatua goffiniana)*. *Behaviour* 1–27 (2018) doi:10.1163/1568539X-00003515.

12. Laumer, I. B., Bugnyar, T., Reber, S. A. & Auersperg, A. M. I. Can hook-bending be let off the hook? Bending/unbending of pliant tools by cockatoos. *Proc. R. Soc. B* **284**, 20171026 (2017).

13. Habl, C. & Auersperg, A. M. I. The keybox: Shape-frame fitting during tool use in Goffin’s cockatoos (Cacatua goffiniana). *PLOS ONE* **12**, e0186859 (2017).

14. Jones, C. G. Conservation management of endangered birds. in *Bird ecology and conservation: A Handbook of Techniques* (eds. Sutherland, W. J., Newton, I. & Green, R. E.) vol. 1 (Oxford University Press, 2004).

15. IUCN/SSC. *Guidelines for Reintroductions and Other Conservation Translocations. Version 1.0. Gland, Switzerland: IUCN Species Survival Commission.* (2013).

16. Baayen, R. *A practical introduction to statistics using R. Analyzing Linguistic Data*. (Cambridge University Press, 2008).

17. Harrison, X. A. *et al.* A brief introduction to mixed effects modelling and multi-model inference in ecology. *PeerJ* **6**, e4794 (2018).

18. Field, A. Discovering statistics using SPSS . Thousand Oaks, CA, US. (2005).

19. Nieuwenhuis, R., te Grotenhuis, M. & Pelzer, B. influence.me: Tools for detecting influential data in mixed effect models. *The R Journal* **4**, 38–47 (2012).

20. Chajewski, M. *rela: Scale item analysis. R package version 4.1*. (2009).

21. Bates, D., Mächler, M., Bolker, B. & Walker, S. Fitting linear mixed-effects models using lme4. *Journal of Statistical Software* **67**, 1–48 (2015).

22. Fox, J. & Weisberg, S. *An R Companion to Applied Regression (Third Edition). R package version 3.0*. (2019).

23. Wickham, H. *ggplot2: elegant graphics for data analysis*. (Springer, 2016).

24. Wilke, C. O. *cowplot: streamlined plot theme and plot annotations for ‘ggplot2’. R package version 3.2.1*. (2019).

25. Quinn, G. P. & Keough, M. J. *Experimental Designs and Data Analysis for Biologists*. (Cambridge University Press, 2002).
